# Clinical efficacy of novel biogenically fabricated titania nanoparticles enriched mouth wash in treating the tooth dentine hypersensitivity: A randomized clinical trial

**DOI:** 10.12669/pjms.41.6.11856

**Published:** 2025-06

**Authors:** Afsheen Mansoor, Emaan Mansoor, Khadim Hussain, Samiullah Khan

**Affiliations:** 1 Afsheen Mansoor, PhD (Medical Microbiology, Dental Materials, Nanotechnology) Associate Professor, Dental Material Sciences Department, School of Dentistry, Shaheed Zulfiqar Ali Bhutto Medical University, Islamabad, Pakistan. Quaid-i-Azam University, Islamabad, 45320, Pakistan; 2 Emaan Mansoor Islamic International Dental College, Riphah International University, Islamabad, Pakistan; 3 Khadim Hussain Statistical Analysist, Islamia University, Bahawalpur, Pakistan; 4 Samiullah Khan, PhD (Medical Microbiology) Associate Professor, Department of Microbiology, Quaid-i-Azam University, Islamabad, 45320, Pakistan

**Keywords:** *Bacillus Coagulans*, Biogenic, Dentin Hypersensitivity, Nanoparticles, Titanium oxide (TiO2 Nps)

## Abstract

**Objective:**

To investigate clinical efficacy of novel biogenically fabricated titania nanoparticles enriched mouth wash in treating dentin sensitivity of patients.

**Methods:**

Tripple blinded randomized clinical trial was conducted at School of Dentistry Islamabad from 6^th^ June 2024 to 6^th^ December 2024 incorporating participants (n = 260) with Group-A (n = 130) and Group-B (n = 130). After attaining informed consent and randomization, Visual Analog Scoring system (VAS Value) was induced to check the Dentin hypersensitivity (DH) of the participants at baseline, after one month and three months follow up. Group-A was given Conventional Mouth wash and Group-B was given Novel-Nanoparticles (Nps) Mouth Wash for total three months to treat DH. The primary outcome was calculated as mean VAS Values for both Groups-A and B after one month and three months. Mann-Whitney-U Test was used for comparing DH between these two groups.

**Results:**

According to trial results, significant difference was obtained between Group -A that used Conventional Mouth wash and Group-B that used Novel- Nps Mouth wash after one month (p = 0.001) and three months (p = 0.002).

**Conclusion:**

DH declination was more prominent in patients that used Novel- Nps Mouth wash with nanoparticles in comparison to participants that used Conventional Mouth wash without any nanoparticles. This concludes that inclusion of biogenically fabricated titania nanoparticles in clinical dentistry could be beneficial in resolving Dentin Hypersensitivity.

## INTRODUCTION

Currently, DH (Dentin Hypersensitivity) is a huge physiologic problem because its prevalence has been increased to 90.0% worldwide affecting age groups ranging between 25-50 years.^1^ Due to this problem, it has become necessary to develop prophylaxis technology on urgent basis for maintaining good quality life of the general population. DH is a well-known painful condition that occurs after exposure and loss of tooth structure because of abrasion, erosion, wear, abfraction and gum recession. This pain is further aggravated when dentine comes in contact with any external stimulus such as evaporative, thermal, tactile, chemical and osmotic pressures.^2^ The mechanism of DH is till now uncertain but hydrodynamic theory of DH is considered as the most fundamental hypothesis that focuses on proper control of dentin fluid movement to avoid any pain and discomfort.^3^ The most common conventional therapeutic agents recommended for providing relief from DH included home use mouthwashes, dentifrices, in-office fluoride based materials, Varnishes, glass ionomer cements, composite resins and lasers.^3^

Conventional materials were rich in fluoride and calcium that would release calcium fluoride which were efficient in reducing DH via mechanical barrier formation. This barrier was helpful in obliterating fluid movement in dentinal tubules thus, reducing pain and DH relief but this effect was limited for shorter duration.^4^ Owing to easy usage, cost efficacy, home application in routine could become sole reason for developing dentifrices and mouthwashes that could relieve DH on larger scale. According to Canadian Advisory Board on Dentin Hypersensitivity (CABDH), Home-Care Approach is advised to be first choice of DH treatment and In-office therapies in Dental clinics would be recommended only in worst scenarios where Home-Care Approaches would fail.^5^

The advent of Nanotechnology has brought robust advancements in clinical dentistry being much easily accessible, cost effective, bio-compatible, ecofriendly, safe and stable in nature.^6^ The TiO2 Nps are considered as best option because of their biocompatibility, antimicrobial activity, mechanical and strength properties. Above all, their optical properties are helpful in improving the tooth color as well which is an added advantage.^7^ Several studies utilized TiO2 Nps obtained from natural sources for treating post-operative DH after certain restorative therapies.^8^,^9^ That might have become possible because of high energy, reactivity and small size of Nps that could have adhered to dentine surface in larger quantities and would have occluded their dentinal tubules, thus resulting in its pain declination and sensitivity.

Moreover, remineralization capacity of Nps is much more prominent as a result of their physicochemical properties as reported in literature.^10^ A study reported that 20% incorporation of Nps in dentifrice reduced DH by 77.4% only if continued for longer duration which is a disadvantage.^4^ Another study reported deduction in DH with help of nano-derived dentifrice for period of two months only.^11^ The desensitizing effects of these products were limited and short lived that proved to be their disadvantage. The Nps are capable of reducing dentine hypersensitivity by blocking the dentinal tubules 10,11 that might have been amplified with the usage of safe, ecofriendly biogenic method of Nps fabrication in comparison to chemical synthesis. To best of our knowledge there is lack of available data on incorporating biogenically derived TiO2 Nps in mouth wash for providing long lasting relief from dentine hypersensitivity in clinical settings. The current study aimed to investigate clinical efficacy of novel biogenically fabricated titania nanoparticles enriched mouth wash in treating dentin sensitivity of patients in a clinical trial.

## METHODS

This clinical trial is an accordance with “CONSORT-2010 GUIDELINES” and was designed as triple blinded, parallel group randomized clinical trial having allocation ratio 1:1.

### Ethical Approval, Clinical Trial registration & study duration:

This study was approved by Ethical Approval Board of Institution under the Ref No: SOD/ERB/2024/36-200 obtained on June 13^th^, 2024. It was registered at Clinical Trials.gov ID No: (NCT06790953) of U.S Protocol Registration and Result System. This study was performed by the Department of Science of Dental Materials at School of Dentistry for period of six months from 6^th^ June, 2024 to 6^th^ December, 2024. All participants were informed about study protocol, its advantages and disadvantages. A written informed consent was obtained from each and every participant who volunteered in this study.

### Inclusion Criteria:

Participants ranging between 20-50 years, good general health but developed dentine sensitivity associated with tooth surface wear during past six months. There was no history of allergy. Participants did not use any mouth wash, dentifrice or medication in last three months. Involved teeth were viable devoid of decay, cracks and fillings with an essential positive response to provoking Air stimuli. These teeth should display visible signs of attrition, abrasion, erosion and abfraction.

### Exclusion Criteria:

Participants that might have developed allergy or used medications during last two weeks or more. Teeth having cracks, decay and fillings were also excluded. Patients who have undergone any periodontal surgery or gross periodontal issues. Participants who used any desensitizing toothpastes/mouthwashes and tooth bleaching agents within two months of trial were also excluded.

### Sample size Calculation:

G-Power Calculator was used to calculate sample size which was 360 with effect size (f=0.17), 95% power and 5% significance having 120 participants in each group. Total sample size considered in this study was 130*3= 390. “Sample size of 130 participants was employed for repeated measures ANOVA, exceeding recommended minimum of 20 participants per group for detecting medium effect sizes (f=0.25) with 80% power at 5% significance, assuming moderate correlation between repeated measures (ρ=0.5). This ensures sufficient statistical power and robustness of results.^12^ Thus, sample size employed in current trial was total 260.^12^ Participants were divided into Group-A (n=130) and Group-B (n=130).

### Sampling Technique:

*S*ampling technique used in this trial was Consecutive Non-Probability where patients that entered OPD (Out Patient Department) of Dental Hospital, School of Dentistry were selected according to inclusion criteria. Trial-autonomous researcher randomly distributed participants in Groups-A and B. The operator, data analyst and participants were kept blinded in this triple blinded study attributed to designated groups. Identical mouth wash bottles were used and were coded with A and B where allocation concealment was also carried out with help of an envelope containing designated codes for each interventional group. Both allocation and trial concealment were performed by trial-autonomous researcher.

### Biogenic Route for TiO2 Nps Synthesis and characterization:

*Bacillus Coagulans strain* (Accession/No: ATCC-®7050™; Catalog/No: 0596-P Micro-biologics; Thermo-Fisher Scientifc; USA) was obtained from National Institute of Health (NIH), Islamabad and then TiO2 Nps were prepared at Dental Materials Department, School of Dentistry, Islamabad.^13^ These TiO2 Nps were investigated for their size, shape, dimensions, phase forms, configuration, surface texture and topological morphology at National Institute of Lasers and Optronics, Islamabad. Characterization revealed 100% pure anatase phase having particle size of about 21.84 nm. These Nps were in form of spherical, small clusters having pure oxygen and Titanium in its elemental composition with highly biocompatible nature.^13^

### Incorporation of TiO2 Nps in the Mouth Wash:

Commercial Sensed Mouth Wash (Gennec Health, Sciences, PVT, LTD, Karachi, Pakistan) was employed where biogenically induced TiO2 Nps were incorporated in it at 20% mass fraction (150g of TiO2 Nps were added in 150ml of Sensed Mouth Wash bottle) and kept in orbital shaker for at least 24 hours.^9^,^14^

### Clinical Trial Procedure:

VAS (Visual analogue Scale) system was employed to investigate Dentine Hypersensitivity (DH). “VAS” system incorporates “100.00” mm scale that represents “0 = Painless condition” and “100 = Worst pain condition”.^15^ Participants having “VAS values” ranging between 30-80 were selected in trial before using mouthwash (baseline values) for both Group-A and Group-B participants. Group-A participants were given Conventional Mouth Wash (Conventional MV) for three months and Group-B participants were given Nps-Novel Mouth Wash (Nps-Novel MV) for three months. VAS values for DH of Group-A and Group-B participants were calculated via exposing their teeth to cold air blast system in triple syringe of Dental unit at PSI 30/pressure ranging between 23±30ºC for one second before using mouth wash. Later on, after giving Conventional MV to Group-A participants for three months, their “VAS values” were calculated with help of VAS scoring tool as VAS-Conventional MV (after one month) and VAS-Conventional MV (after three months). On other hand, “VAS values” were calculated with help of VAS scoring tool as VAS-Nps Novel MV (after one month) and VAS-Nps Novel MV (after three months) for Group-B participants where Nps-Novel MV was induced. Conventional and Novel MV was recommended twice a day (sixty seconds) at least for three months. The MV name was hidden from participants of this study where they were advised to maintain proper oral-hygiene, avoid erosive edibles and other dental treatments for preventing any sort of Bias.^16^

### Statistical Analysis:

SPSS Software Version 26.00 was employed for calculating Means, Standard Deviations and Standard Error values. Pair wise comparisons were carried out with help of One-way ANOVA. Independent T test was employed for comparison of VAS scoring between Conventional and Novel MV users. Confidence level was kept at 95.00% depicting significance level of about p-value ≤ 00.05.

## RESULTS

**Fig.1:**
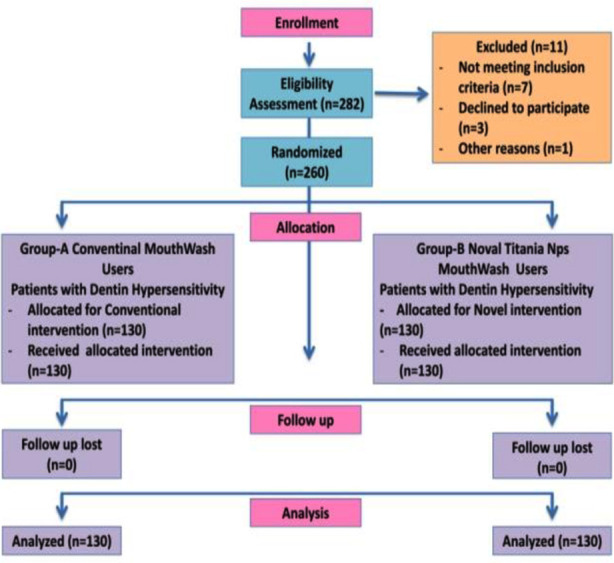
CONSORT flow chat depicting the allocation of the participants in the current trial.

**Fig.2:**
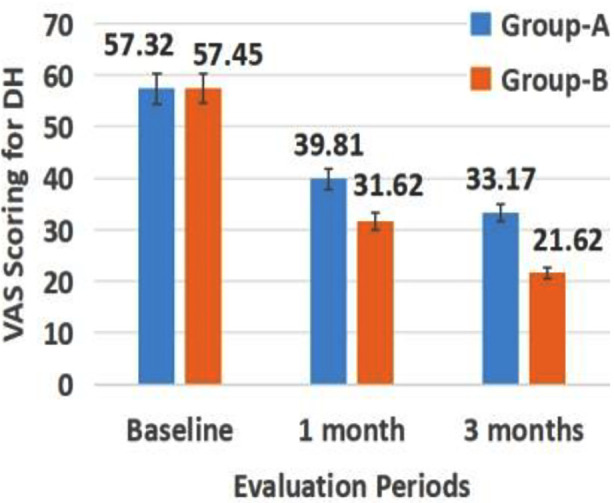
Descriptive statistics of VAS scoring measurement in Group-A and Group-B at baseline, after one month and three months.

Participants enrolled in current trial were 282 of which seven participants did not meet the inclusion criteria, three participants declined to participate and one participant did not give any reason (Fig.1). Group-A comprised of 73 males and 57 females having mean VAS scoring of 57.32 + 12.05 for DH at baseline whereas Group-B constituted of 69 males and 61 females with mean VAS scoring of 57.45 + 12.06 for DH at baseline. Descriptive statistics in terms of mean, standard deviation of VAS scoring in Group-A using Conventional MV at baseline, after one month and three months and Group-B using Nps Novel MV at baseline, after one month and three months was illustrated in Fig.2. The declination in VAS Scoring measurements was observed in Group-A after using Conventional MV at baseline and one month (p=0.001), baseline and three months (p=0.002), and at one month and three months (p=0.004) (Table-I). Group-B using Novel Nps MV displayed reduction in VAS Scoring measurements at evaluation time periods of baseline and one month (p=0.001), baseline and three months (p=0.002), and at one month and three months (p=0.004) (Table-I). Thus, reduction in DH was confirmed in both Groups-A and B.

**Table-I:** ANOVA Pair Wise Comparison of VAS scoring in intergroup measurements of Group-A and Group-B at baseline, after one month and three months.

Groups	Comparison of VAS scoring between different Groups-At different Evaluation Periods	Mean Difference with Standard Error (S.E)	95% Confidence Interval		ANOVA value	P-value
			Lower Limit	Upper Limit		
Group-A	Comparison of VAS scoring of participants using Conventional MV at Baseline and after 1 month	17.51 (0.71)	16.02	18.99	291.52	0.001
	Comparison of VAS scoring of participants using Conventional MV at Baseline and after 3 months	24.15 (0.61)	22.43	25.87	291.52	0.002
	Comparison of VAS scoring of participants using Conventional MV after 1 month and after 3 months	6.64 (0.51)	5.41	7.87	291.52	0.004
Group-B	Comparison of VAS scoring of participants using Novel Nps MV at Baseline and after 1 month	25.83 (0.74)	24.02	27.64	291.52	0.001
	Comparison of VAS scoring of participants using Novel Nps MV at Baseline and after 3 months	35.83(0.76)	34.02	37.64	291.52	0.002
	Comparison of VAS scoring of participants using Novel Nps MV after 1 month and after 3 months	10 (0.81)	10.00	10.00	291.52	0.004

### Statistical analysis:

Statistical analysis by applying t-test revealed statistically significant reduction in DH between Group-A Conventional MV and Group-B Nps-Novel MV Groups-A at one month (t = 7.305, p < 0.001). Group-B Novel MV group reported lower VAS scores (M = 31.62) than Group-A Conventional MV group (M = 39.81), with mean difference of 8.19 points (p value = 0.001). After three-months, t-test also indicated statistically significant reduction in DH (t = 12.422, p < 0.001). Group-B Nps-Novel MV group showed significantly lower VAS scores (M = 21.62) than Group-A Conventional MV group (M = 33.17), with larger mean difference of 11.55 points (p value = 0.002). Group-B Nps-Novel MV intervention consistently demonstrated greater efficacy in reducing DH compared to Group-A Conventional MV approach, with substantial and clinically relevant effects evident after both one and three months (Table-II).

**Table-II:** Independent T test displaying reduction in DH by comparing VAS scoring between Group-A and Group-B at different evaluation periods.

Groups	Comparison of VAS scoring between Conventional MV users and Novel Nps MV at different Evaluation Periods	Mean Difference with Standard Error (S.E)	95% Confidence Interval		t	P-value
			Lower Limit	Upper Limit		
Comparison of Group-A and Group-B after 1 month	Comparison of VAS scoring of participants using Conventional MV and Novel Nps MV after 1 month	8.192 (1.12)	5.98	10.40	7.30	0.001
Comparison of Group-A and Group-B after 3 months	Comparison of VAS scoring of participants using Conventional MV and Novel Nps MV after 3 months	11.55 (0.93)	9.72	13.39	12.42	0.002

## DISCUSSION

The novelty of current research lies in hall mark that biogenically procured TiO2 Nps have never been used in conventional mouth wash in order to treat DH. Additionally, these Nps were employed in lowest concentration (20%) in Nps-Novel MV used in this research where this percentage was well effective in reducing DH both after one month and three months as compared to Conventional MV. This confirms that only 20% Nps-Novel MV was potent enough in treating DH of patients involved in current clinical trial after using it for shorter duration.

Several reported studies displayed reduction in DH with nano-fortified adhesives^8^,^9^ and dentifrices^17^,^18^ after using them for longer time duration of more than three months in comparison to Novel TiO2 Nps mouth wash utilized in current study that brought amplified declination in DH just after one month and completely diminishing DH after three months. This can be taken as strength of this clinical trial where nano size of these Nps might have possibly played a significant role in eliminating DH entirely.

Literature reported that TiO_2_ nanoparticles derived from natural biogenic sources have depicted enhanced biocompatibility, safety, antimicrobial activity with enhanced properties.^7^,^13^,^19^,^20^ TiO2 Nps originated from *Bacillus Coagulans*^13^ induced in Novel Nps MV of current study revealed unique physico-chemical features including ideally small size, spherical shape and smooth texture that might have absorbed into exposed dentinal tubules in large amounts thus, occluding them and diminishing effects of DH. Moreover, anatase form of these Nps is reactive phase that could have been sole reason of forming quick protective smear layer on Dentin’s outer surface. This protective layer might serve as future shield in reducing DH.

The significant antimicrobial activity of these TiO2 Nps used in current trial might also be effective in declining adherence of biofilm forming bacteria on surfaces of dentin in turn preventing dental caries and other oral diseases. Therefore, future studies are required to utilize naturally procured Nps in therapeutic agents in order to protect tooth surfaces from secondary caries attack. Mostly, quality life of any individual is compromised due to Dentin hypersensitivity. It is common problem whose incidence has increased up to 74% and prevalence up to 98% which got extremely uncomfortable for individuals suffering from this condition. Currently, DH occurs as a result of natural forces occurring in oral cavity such as wear, attrition, abrasion, and erosion that can’t be stopped but could be suppressed or treated otherwise.^21^ Thus, further studies are required to induce biogenic Nps in dentifrices and mouth washes for preserving tooth dentin surfaces from such masticatory forces.

Dentin-Pulp complex also gets thinner over time exposing surface of dentin eventually increasing DH^22^, that could be reduced only with help of natural nano-fortified tooth pastes and mouth washes in order to form protective smear layer on dentin surface thus treating DH. With advent of nanotechnology, multiple oral health related issues have been resolved in this epic including DH, periodontal diseases, gum recession problems and cariogenicity. Previous studies reported that commercially available nanoparticles have been incorporated in tooth pastes which reduced DH to some extent after using them for longer duration and in larger quantities.^11^,^23^,^24^ That might pose harmful effects on dentin surface due to their compromised safety and compatibility with oral tissues that might have resulted because of their prolonged utilization in large amounts. Thus, it is recommended to employ naturally fabricated Nps in vast dental preventive agents because of their enhanced stability, bio compatibility and safety that makes them ideal candidates for oral applications. The strength and importance of the current trial prevails in the fact that the Nps-Novel MV utilized in this study displayed long lasting effects of this new product in treating the dentin hypersensitivity in the shorter time duration that is an added advantage of this latest Nps-Novel MV. More studies are required to prepare TiO_2_ Nps from certain natural resources available in environment and their utilization in clinical dentistry for remineralization purposes, in order to avoid wear, abrasion, abfraction, erosion and roughness in the natural tooth structure caused by the normal masticatory and brushing forces.

### Limitations

Future studies are required to treat Dentin Hypersensitivity with more naturally procured nanoparticles. Modifications are required in the concentrations to treat the DH within few weeks instead of using it for months.

## CONCLUSION

The current trial concluded that declination in DH was more quick and evident in patients that utilized Novel- Nps Mouth wash having nanoparticles as compared to participants that used Conventional Mouth wash without any nanoparticles. This shows that inclusion of biogenically fabricated titania nanoparticles in clinical dentistry could be beneficial in resolving Dentin Hypersensitivity.

### Authors Contribution:

**AM and SK:** Conceptualization, Writing - Review & Editing and Critical Review.

**AM and EM:** Methodology, Data Collection, Data Processing and Writing - Original Draft.

**AM and KH**: Data Analysis & Interpretation.

**SK:** Supervision, Accuracy or Integrity of this study.
